# Laparoscopic Repair of Congenital Diaphragmatic Hernia in Adults

**DOI:** 10.1155/2016/9032380

**Published:** 2016-12-15

**Authors:** Sanjay Kumar Saroj, Satendra Kumar, Yusuf Afaque, Abhishek Kumar Bhartia, Vishnu Kumar Bhartia

**Affiliations:** ^1^Minimal Access Surgery, Institute of Medical Sciences, BHU, Varanasi, India; ^2^General Surgery, Institute of Medical Sciences, BHU, Varanasi, India; ^3^AIIMS, New Delhi, India; ^4^CMRI, Kolkata, India

## Abstract

*Background, Aims, and Objectives*. Congenital diaphragmatic hernia typically presents in childhood but in adults is extremely rare entity. Surgery is indicated for symptomatic and asymptomatic patients who are fit for surgery. It can be done by laparotomy, thoracotomy, thoracoscopy, or laparoscopy. With the advent of minimal access techniques, the open surgical repair for this hernia has decreased and results are comparable with early recovery and less hospital stay. The aim of this study is to establish that laparoscopic repair of congenital diaphragmatic hernia is a safe and effective modality of surgical treatment.* Materials and Methods.* A retrospective study of laparoscopic diaphragmatic hernia repair done during May 2011 to Oct 2014. Total *n* = 13 (M/F: 11/2) cases of confirmed diaphragmatic hernia on CT scan, 4 cases Bochdalek hernia (BH), 8 cases of left eventration of the diaphragm (ED), and one case of right-sided eventration of the diaphragm (ED) were included in the study. Largest defect found on the left side was 15 × 6 cm and on the right side it was 15 × 8 cm. Stomach, small intestine, transverse colon, and omentum were contents in the hernial sac. The contents were reduced with harmonic scalpel and thin sacs were usually excised. The eventration was plicated and hernial orifices were repaired with interrupted horizontal mattress sutures buttressed by Teflon pieces. A composite mesh was fixed with nonabsorbable tackers. All patients had good postoperative recovery and went home early with normal follow-up and were followed up for 2 years.* Conclusion.* The laparoscopic repair is a safe and effective modality of surgical treatment for congenital diaphragmatic hernia in experienced hands.

## 1. Introduction 

Congenital diaphragmatic hernia typically presents in childhood. However, its clinical manifestation and diagnosis in adults are extremely rare. They occur due to failure of the development of muscular diaphragm, which leads to displacement of the abdominal component into thorax. The diagnosis of congenital diaphragmatic hernia is based on clinical investigation and is confirmed by plain X-ray film barium study and computed tomography scans.

There are four types of congenital diaphragmatic hernia: posterolateral hernia of Bochdalek, parasternal hernia of Morgagni-Larrey, eventration of diaphragm, and peritoneal-pericardial hernia. Bochdalek hernia is the most common type of congenital diaphragmatic hernia, which was first described by Bochdalek in 1848 [1]. It develops due to the failure of closure of the posterolateral aspect of pleuroperitoneal canal, which takes place between 8 and 10 weeks of gestation. As the left canal closes later than the right, it occurs on the left side in 85% of cases [2].

Eventration of the diaphragm is a congenital anomaly consisting of failure of muscular development of part or all of one or both hemidiaphragms [3]. Clinically, eventration of diaphragm refers to an abnormal elevation of one leaf of an intact diaphragm as a result of paralysis, aplasia, or atrophy of varying degrees of muscle fibers [4]. In some cases, it may be difficult or impossible to distinguish from diaphragmatic paralysis. Complete eventration almost invariably occurs on the left side [5] and is rare on the right [6]. There are very few cases of the right-sided diaphragmatic hernia reported in adults in the literature.

It may be associated with other congenital anomalies. The prevalence of Bochdalek hernia is one in 2,200 births [7] and only 5–10% of them remain undetected in childhood, which presents in adults [8].

Surgery is indicated for symptomatic [6] as well as asymptomatic patients who are fit for surgery [4, 7, 9]. It can be done by laparotomy, thoracotomy, thoracoscopy, or laparoscopy [10, 11]. With the advent of minimal access techniques, the open surgical repair for this hernia has decreased. The results of thoracoscopy and laparoscopy in such cases have been found to be comparable. Laparoscopic repair helps in delineating clear anatomy, working space, early recovery, and return to home and work. Campos and Sipes [12] did the first laparoscopic repair of diaphragmatic hernia in 1991. Kuster et al. followed it in 1992 [13]. Till now only small case series and case report are available in the literature. The aim of this study is to establish that laparoscopic repair of congenital diaphragmatic hernia is a safe and effective modality of surgical treatment in experienced hands.

## 2. Material and Methods

We present a retrospective study of diaphragmatic hernia repair done laparoscopically during period of May 2011 to Oct 2014. Total *n* = 13 (M/F: 11/2) cases of diaphragmatic hernia were treated (Table 1). Average age of presentation was 36 years (28–54 years). One patient was having right-sided eventration of the diaphragm, while another 12 were presented with left-sided eventration. Bochdalek hernia (BH) was found in 4 patients while the remaining 9 patients were having eventration of the diaphragm. Most patients were having a complex of clinical features.

Abdominal pain (Table 2) and discomfort were the most common presenting complaints in 10 (76.9%) patients followed by dysphagia and GERD in 7 (23%) patients and respiratory distress and cough in 3 (23%) patients. One patient presented with features of small bowel obstruction while one patient was clinically asymptomatic. On clinical examination there was decreased breath sound on left lower chest and also on the right side in a right-sided eventration of the diaphragm. Routine blood investigations were normal. X-ray of the chest showed an elevated left hemidiaphragm and pleural effusion in the left sided diaphragmatic hernia while the elevation of the right hemidiaphragm appeared in the right sided. CT thorax and abdomen were used as a diagnostic modality showing the splenic flexure of the colon and small intestine in the left chest causing mediastinal shift to right. Stomach, 1st, and 2nd part of the duodenum were grossly distended in an obstructed diaphragmatic hernia (Figure 5). In right-sided diaphragmatic hernia there was a large protuberance of liver pushing the right lung (Figure 3).

**Figure 1:**
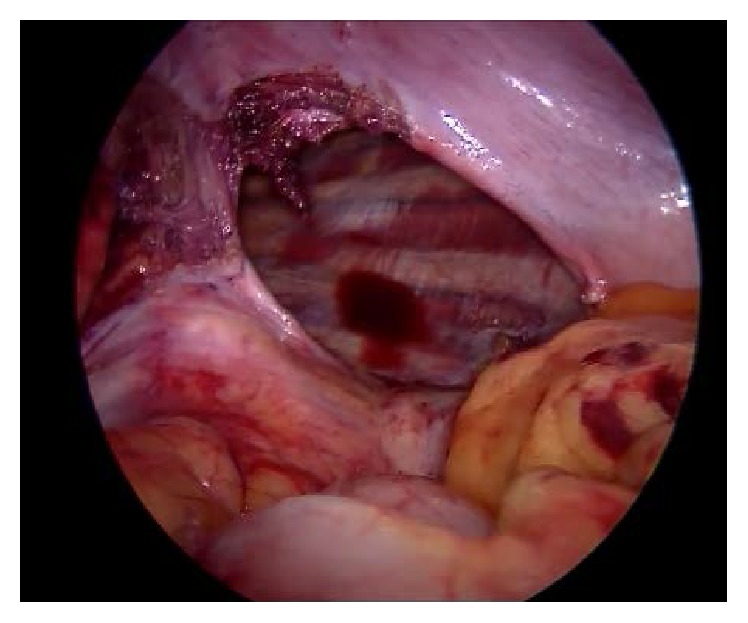
Large Bochdalek hernia on left side.

With a preoperative diagnosis of diaphragmatic hernia, the laparoscopic repair was planned. The patient was placed in 30° reverse trendelenburg position with a sand bag under left lower chest. After insufflations of abdomen, five ports were placed. Presence of diaphragmatic hernia (Table 3) was confirmed, and 4 cases of Bochdalek hernia (BH), 8 cases of left eventration of diaphragm, and one case of right-sided eventration of the diaphragm were included in this study. Largest defect found on the left side was 15 × 6 cm (Figure 1) and on the right side it was 15 × 8 cm. Stomach, small intestine, transverse colon, and omentum were found to be in the hernial sac on left side.

**Figure 2:**
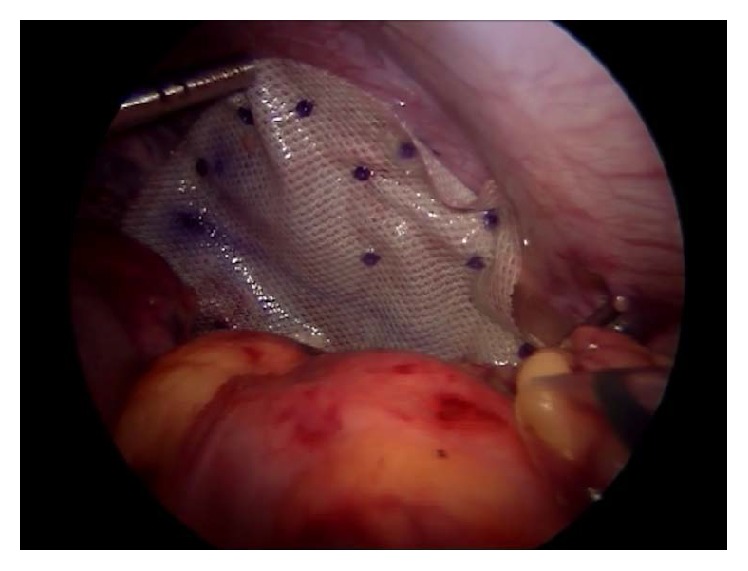
Composite mesh fixed with tackers.

**Figure 3:**
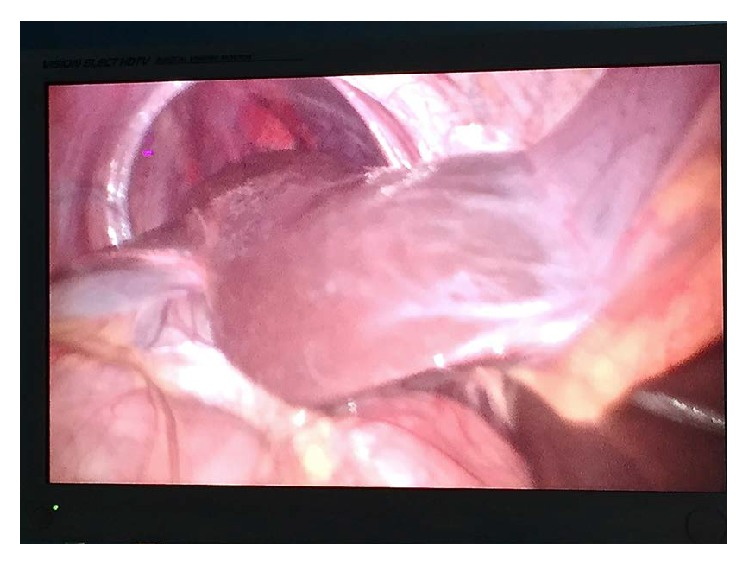
Right-sided eventration of diaphragm content as liver protuberance.

The contents were reduced with the help of harmonic scalpel dissection and however thin sacs were excised. After reduction of contents, hernial orifice was repaired with Polypropylene—1/0 (Ethibond/Prolene). Interrupted horizontal mattress sutures were placed buttressed by Teflon pieces. A 20 × 15 cm (Figure 2) composite mesh (Parietex) was placed over the defect fixed with nonabsorbable tackers. Diaphragmatic plication with (Ethibond) mesh placement was a procedure done in all 9 cases of eventration of the diaphragm.

On the right-sided diaphragmatic hernia the content was liver protuberance, which was reduced with limited conversion procedure compared to the plication of diaphragm with mesh placement being done. Average operating time was 145 mins (110–180 mins). Patients were ambulated and next day liquid diet was given. A postoperative chest X-ray was done on postoperative day one. The average hospital stay was 4 days (2–6 days) and patients were discharged on PPI and analgesic. All patients were followed up for 2 years and one patient was lost in follow-up after one year. All of them were having improvement in clinical symptoms while there was persistent dull aching pain in 3 patients; dyspepsia and fullness in 4 patients were having respiratory distress.

## 3. Discussion

Congenital diaphragmatic hernia presents in different ways in adults and pediatric age group. Cyanosis and respiratory distress are predominant features in neonates and infancy, while in adults it presents with chest pain, difficulty in breathing, abdominal pain, and sometimes intestinal obstruction [14]. Some cases may remain asymptomatic and it may be due to the occlusion of diaphragmatic defect by the intra-abdominal viscous [15]. Two-thirds of asymptomatic cases have been found to be on the right side and it is mainly because of the liver which prevents herniation of other organs [16].

**Figure 4:**
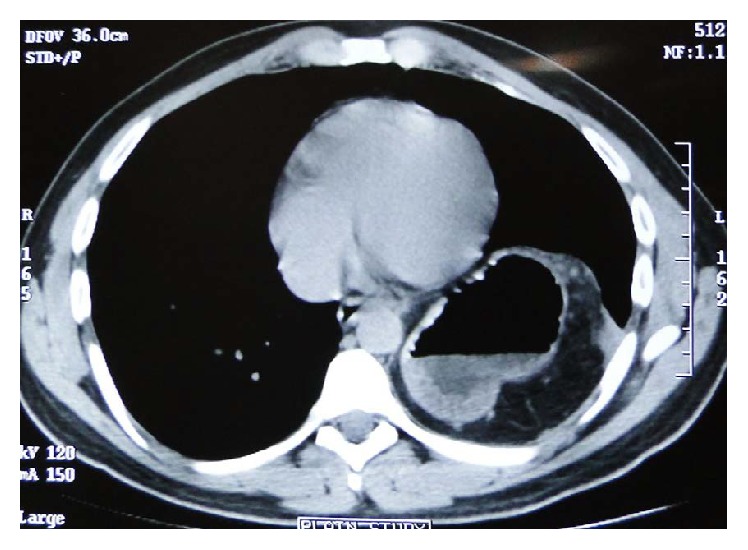
CT scan showing stomach herniation in left Bochdalek hernia.

**Figure 5:**
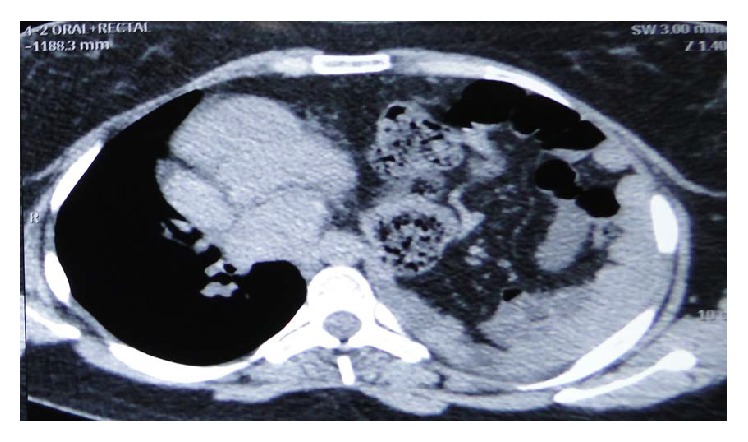
CT scan showing small intestine herniation in left Bochdalek hernia.

In congenital diaphragmatic hernia various intra-abdominal organs can herniate into the thorax. Organs that commonly herniate are stomach (Figure 4), ileum, colon, and spleen and on the right side liver and right kidney may also herniate along with the bowel loops. A left side Bochdalek hernia may be associated with lung hypoplasia, extralobar sequestration, malrotation of midgut, and cardiac defects and on the right side, it is often associated with hypoplasia of the right lobe of liver [17].

Bochdalek hernia is a rare hernia in adults, so misdiagnosis is common. Inappropriate intervention, such as chest tube placement, can occur in the cases of misdiagnosis [18–21]. The delay in diagnosis may also result in strangulation and death [22]. CT scan is the most accurate imaging modality for the diagnosis and evaluation of the contents of this hernia, especially when it is small [23, 24]. About 38% of these adults are misdiagnosed as pleural effusion, empyema, lung cyst, and pneumothorax when CT scan is not done [25]. One of our patients was also diagnosed as a case of pleural effusion before CT scan was done. The other advantage of the CT scan was here to detect the presence of spleen as one of the contents and helped in cautious handling of this friable organ to prevent the catastrophe [26]. MRI is an alternative diagnostic modality.

In the laparoscopic repair of Bochdalek hernia visualisation and working space is excellent (Table 4) [27]. One difficulty faced is that the contents tend to go back in the thorax because of the positive intra-abdominal pressure due to pneumoperitoneum and it is overcome by holding it back in the abdomen with a grasper. In incarcerated cases the part of diaphragm forming the neck of the hernia may need to be opened to reduce the contents. The hernial sac in Bochdalek hernia is present in only 10–15% of patients and it can be excised or left as such. The dissection of the sac may also lead to pleural injury [28]. A study was performed in which a CT scan was done on postoperative day 30 to see what happens to the left over hernial sac, and it was seen that the sac had disappeared [29].

The closure of the defect can be done by different methods. When the defect is small it can be simply sutured closed, but when it is large (>10 cm square) it will need a prosthetic reinforcement [9, 30]. Sufficient evidence favoring any particular type of mesh is lacking [31]. In our practice, we close the defect with horizontal mattress over the Teflon sheets with the nonabsorbable sutures. And then we put a large piece of composite mesh to cover the whole hemidiaphragm. The advantage of Teflon sheet is that it prevents the cutting of the diaphragmatic edges when the tension is high during approximation in large defects. Mesh repair was used to strengthen the weak diaphragm.

## 4. Conclusion

CDH in adults is an uncommon form of diaphragmatic hernia. The laparoscopic repair is a safe and effective modality of surgical treatment in experienced hands.

## Figures and Tables

**Table 1:** Demographic profile of patients.

Number of patients *N* = 13	
Average age (yrs)	36 yrs
Range of age	28 yrs to 54 yrs
Sex	M : F = 11 : 2
Left sided	12
Right sided	1
Associated factors	
Trauma	7
Congenital	5
Pregnancy	1
Other associated anomalies	Nil

**Table 2:** Clinical manifestation.

Clinical features	Number of patients	Percentage (%)
Abdominal pain	10	76.9
Respiratory distress	3	23%
Cough	2	15.3%
Vomiting	2	15.3%
Intestinal obstruction	1	7.69%
Strangulation	0	—
Asymptomatic	1	7.69%
GERD	7	53.8%
Dysphagia	4	30.77%

**Table 3:** Hernial description.

*Pathology*		
Type of defect	Bochdalek hernia = 4	
	Eventration of left side diaphragm = 8	
	Eventration of right side = 1	
Size of defect	Largest = 15 × 8 cm	Smallest 8 × 8
Content of defect	Right side = liver	
	Left side = stomach = 4	
	Colon = 3	
	Stomach with spleen = 1	
	Omentum = 2	

**Table 4:** Treatment option and complications.

*Treatment*	
Laparoscopic	10
Open repair	1
Plication with mesh repair	10
Mesh repair	3
*Postoperative complication*	
Persistence of pain	3
Dyspepsia	4
Respiratory distress	2
